# Synonymous Mutations of Porcine *Igf1r* Extracellular Domain Affect Differentiation and Mineralization in MC3T3-E1 Cells

**DOI:** 10.3389/fcell.2020.00623

**Published:** 2020-07-09

**Authors:** Chunli Wang, Siyao Wang, Songcai Liu, Yunyun Cheng, Hongwei Geng, Rui Yang, Tianqi Feng, Guanhong Lu, Xiaotong Sun, Jie Song, Linlin Hao

**Affiliations:** College of Animal Sciences, Jilin University, Changchun, China

**Keywords:** synonymous mutations, IGF-1R, cell differentiation, osteoblast mineralization, protein conformation

## Abstract

Owing to the wide application of miniature pigs in biomedicine, the formation mechanism of its short stature must be elucidated. The insulin-like growth factor 1 receptor (IGF-1R), which receives signals through the extracellular domain (ECD) binding with ligands, is crucial in regulating cell growth and bone matrix mineralization. In this study, two haplotypes of *Igf1r* with four synonymous mutations in the coding sequences of IGF-1R ECD between large pigs (LP) and Bama pigs (BM) were stably expressed in the *Igf1r*-knockout MC3T3-E1 cells and named as MC3T3-LP cells (LP group) and MC3T3-BM cells (BM group), respectively. IGF-1R expression was lower in the BM group than in the LP group both in terms of transcription and translation levels, and IGF-1R expression inhibited cell proliferation. In addition, IGF-1R expression in the BM group promoted early-stage differentiation but delayed late-stage differentiation, which not only suppressed the expression of bone-related factors but also reduced alkaline phosphatase activity and calcium deposition. Moreover, different haplotypes of *Igf1r* changed the stability and conformation of the protein, further affecting the binding with IGF-1. Our data indicated that the four synonymous mutations of IGF1R ECD encoded by affect gene transcription and translation, thereby further leading to differences in the downstream pathways and functional changes of osteoblasts.

## Introduction

Miniature pigs possess many similarities with humans in terms of anatomical, morphological, and physiological characteristics. Hence, miniature pigs have invaluable advantages as biomedical animal models (Liu et al., 2010). With the development and utilization of miniature pig models, current studies focus on xenotransplantation and model construction. However, a systematic study on the formation mechanism of miniature pig dwarfism is lacking (Cheng et al., 2018; Cao et al., 2019). The genetic background of miniature pigs and the mechanism of dwarfism are the prerequisites of scientific research using miniature pig models. Therefore, the formation mechanism of miniature pigs must be elucidated. In China, the Bama Xiang pig is a well-known miniature pig breed from Guangxi Zhuang Autonomous Region. It is characterized by highly inbred, stable heredity and mini-body size (the mean body weight of adults is nearly 40 kg) (Cheng et al., 2016; Yang et al., 2018). The Large White pig also called Yorkshire pigs, is considered the most representative of large pigs because of its large body size and high growth rate (mean body weight of adults is almost 250 kg) and is considered as the most typical representative of large pigs. The contrast in the size and bodyweight of these two breeds makes them an ideal model for comparing the differences in body size between miniature and large pigs. Moreover, bone size (bone mass, volume) and bone growth are regarded as important indicator of body size in mammals (Ruff, 2003; Nieves et al., 2005; Schlecht et al., 2015). Cellular signaling pathways controlled by growth factors and hormones are also believed to influence bone size and bone growth (Efstratiadis, 1998).

The insulin-like growth factor-1 receptor (IGF-1R) is a typical receptor tyrosine kinase that regulates embryonic and postnatal growth (Efstratiadis, 1998; Siddle, 2011). An intragenic IGF-1R deletion that was identified in a patient presented short stature (Harmel et al., 2013). IGF-1R signaling, which is activated by IGF-1, plays an essential role in cell growth and development, as well as in bone formation via osteoblast-mediated bone mineralization (Fang et al., 2019). IGF-1R is also involved in bone matrix mineralization. Studies using osteoblast culture systems have shown that IGF-1R, by binding to IGF-1, enhances bone matrix production and stimulates the proliferation and differentiation of osteoblasts (Canalis, 1993; Birnbaum et al., 1995; Wang et al., 2015). IGF-1R signaling activates autophagy, which is necessary for stimulating early osteoblast differentiation, at the early stage of differentiation by activating AMPK (Xi et al., 2016).

IGF-1R exhibits different protein expression levels in the liver and muscle tissues of large and miniature pigs (Cheng et al., 2016). A previous study screened nine strong linkage synonymous mutations in the coding sequence (CDS) of IGF-1R between large and miniature pigs, four of which were found in the extracellular domain (ECD), whereas five were located in the intracellular domain (ICD) (Changhong et al., 2018). As a ligand-dependent receptor, mutations in IGF-1R ECD can potentially affect its biological function. A clinical research indicated that mutations in *Egfr* are associated with *Igf1r* variants in female patients with lung adenocarcinoma (Liu et al., 2016). A study showed that the mutant of V599E-IGF-1R ECD interferes with the receptor’s transport processes, thereby eliminating the processing of pro-receptors and localization of the plasma membrane (Wallborn et al., 2010). However, most studies presently focus on the missense mutations of *Igf1r* (Wallborn et al., 2010; Liu et al., 2016). A systematic functional research on synonymous mutations is lacking.

Changes in synonymous codons that do not alter the final protein sequence were previously regarded as silent mutations without any functional consequences. Most recent evidence shows that synonymous mutations are shaped by evolutionary selection and affects other aspects of protein biogenesis (Chaney and Clark, 2015). Advances in synthetic biology have provided researchers with new methods for understanding the diverse roles of synonymous variations (Hunt et al., 2014). Synonymous codon usage affects multiple steps of transcription and translation processes, including regulation of speed and accuracy of the translation, co-translational folding, protein post-translational modifications, secretion, and expression levels (Plotkin and Kudla, 2011). Therefore, exploring the functions of synonymous mutations may be the key to uncovering the influence mechanism of the correlation between gene polymorphisms and phenotypes.

**FIGURE 1:**
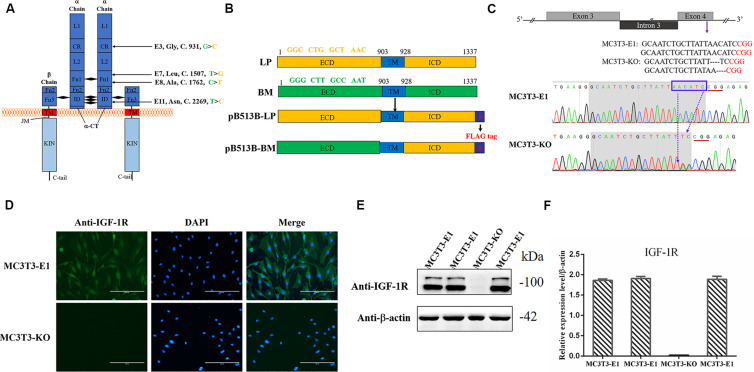
Generating *Igf1r*-Knockout MC3T3-E1 Clones. **(A)** Four SNPs were located in the coding sequence of the *Igf1r* gene ECD between miniature (green) and large (yellow) pigs. **(B)** The full-length *Igf1r* of large pigs (LP) and Bama Xiang pigs (BM) were shown in the top line. The sequences were inserted into the pB513 vector between the *Eco*RI and *Xho*I sites and respectively named as pB513B-LP (with the CDS of full *Igf1r* of Large White pigs) and pB513B-BM (with the CDS of IGF-1R ECD of Bama Xiang pigs and IGF-1R ICD of Large White pigs). TM: transmembrane region (blue), F: FLAG tag (purple). **(C)** Schematic illustration and DNA sequence map showing the position of sgRNA target site. The target sequence and PAM sequence were highlighted by the gray background and red underline, respectively. **(D)** Immunostaining of IGF-1R (Green) and DAPI (Cyan) in MC3T3-E1 and MC3T3-KO cells. **(E)** The protein expression levels of IGF-1R in MC3T3-E1 and MC3T3-KO cells were analyzed by western blot. **(F)** Quantification of the **(E)** western blot results.

Although the growth-related traits of Angus cattle have been proved to be related to a synonymous mutation of *Igf1r* (Szewczuk et al., 2013), the question of whether the synonymous mutations in *Igf1r* can affect the body size traits in pigs remains unclear. Moreover, the potential functions of these synonymous mutations have yet to be recognized. In the present study, we focused on four single nucleotide polymorphisms (SNPs) of IGF-1R ECD previously screened from pigs of different body size traits (Figure 1A and Table 1) to confirm the effects of synonymous mutations on the differentiation and mineralization of osteoblasts. We further clarified the molecular mechanism of bone development to determine the effects of *Igf1r* synonymous mutations on the formation of body shape traits. We expected to provide new evidence clarifying the roles of IGF-1R in the formation mechanism of miniature pigs.

**TABLE 1:** SNPs parameters of IGF-1R gene ECD in Bama Xiang pigs and large pigs.

SNP number	Gene		mRNA	Protein
	Position	Exon	Allele change	Position
rs338724264	g.267380	3	G/C	301
rs325909655	g.286723	7	T/G	493
rs337838116	g.288540	8	C/T	578
rs326728191	g.296721	11	T/C	747

The linkage effects of these synonymous mutations may be involved in the formation of body size in miniature pigs. The present study explored the functions of potentially valuable synonymous mutations and provided a theoretical basis for the formation of body size in miniature pigs. According to the results, we indeed observed differences of IGF-1R at both mRNA and protein levels between the two haplotypes of IGF-1R from large and miniature pigs. Furthermore, these cellular and biochemical alterations affected the stability of IGF-1R and its ability to bind its ligand. Importantly, our results reveal that four synonymous mutations of IGF-1R contribute to the consequent changes in IGF-1R signaling and cellular functions observed in the proliferation, differentiation, and mineralization of osteoblasts.

## Materials and Methods

### Construction of sgRNA and PiggyBac Vectors

The sgRNA vector was constructed as follows: One sgRNA of *Igf1r* in exon 4 was designed by the Crispr/cas9 sgRNA prediction website^1^, and the PX458 knockout vectors containing the sgRNA were constructed (Zafra et al., 2018). The sgRNA forward primer was 5′-CACCGCAATCTGCTTATTAACATC-3′, whereas the reverse primer was 5′-AAACGATGTTAATAAGCAGATTGC-3′. The PiggyBac vector was constructed as follows: Two fusion genes were made using the CDS of *Igf1r* ICD to splice the ECD of the Large White pig and Bama Xiang pig. Two fusion genes contained the FLAG tag sequence and enzyme recognition sequences, which were then synthesized by Jilin Comate Bioscience Co., Ltd (China), and the FLAG tag sequence was added to C-terminal tail of IGF-1R (Figures 1A,B and Table 1). The fusion genes were successfully connected to the pB513B-Puro vector (MiaoLingBio, China). These genes were named pB513B -LP (with the CDS of full *Igf1r* of Large White pigs) and pB513B-BM (with the CDS of *Igf1r* ECD of Bama Xiang pigs and *Igf1r* ICD of Large White pigs) (Figure 1B).

### Cell Culture

Given that the osteoblast cell lines of pigs are difficult to obtain, a mouse osteoblast cell line (MC3T3-E1 cells), which was obtained from the Hospital of Stomatology Jilin University, was selected. The IGF-1R amino acid sequences in mice (NP_034643.2) were found to be 95% similar to those in humans (NP_000866.1) and pigs (NP_999337.1) according to NCBI^2^. Moreover, a certain amount of IGF-1R was detected in MC3T3-E1 cells (Joung et al., 2013). The cells were cultured in Dulbecco’s minimal essential medium (DMEM) (Hyclone, United States) supplemented with 10% heat-inactivated fetal bovine serum and 1% streptomycin/penicillin (Gibco, United States) in an incubator with 5% CO_2_ at 37°C. At 80% cell confluence, the culture medium was changed to a differentiation medium that contained 50 mg/mL ascorbic acid (Sigma, United States) and 10 mmol/L β-glycerophosphate (Sigma, United States) (Zheng et al., 2018).

### *Igf1r* Knockout in MC3T3-E1 Cell Lines

Exactly 30 μg of PX458-sgRNA vectors were transfected into MC3T3-E1 cells by using the BTX-ECM 2001 Electroporation system (United States). Two days after electroporation, the cells expressing the GFP fluorescent protein were separated via flow cytometry for further culture. A few days later, the monoclonal cells were selected and seeded in 24-well plates. When the cells became confluent, NP40 lysis buffer was used to lyse a few cells. The lysate was used as the PCR template for genotyping to identify the knockout activity, and IGF-1R antibody was used for Western blot and immunofluorescence assays further to verify the expression of IGF-1R in MC3T3-E1 cells.

### Generation of Two Recombinant Cell Lines

Exactly 30 μg of pB513B-LP and pB513B-BM vectors and 12 μg PiggyBac transposase vector were successively co-transfected into MC3T3-KO cells using the BTX-ECM 2001 electroporation system. Two days after electroporation, the cells transfected with pB513B-puro vectors were screened with puromycin (3 μg/ml). After 9 days, individual cell clones were selected and seeded in 24-well plates. When the cells became confluent, NP40 lysis buffer was used to lyse a few cells. Genotyping was performed using lysate as the PCR template. The integrated *Igf1r* gene copy number per genome was determined via quantitative real-time PCR (q-PCR). q-PCR was performed using SYBR Select Master Mix (Roche). The primers used are listed in Table 2. For each sample, 100 ng of the DNA template was amplified in PCR reactions on an ABI PRISM 7900HT thermocycler (Applied Biosystems, United States). All samples were performed in triplicate, and each quantification data represented an average of at least three measurements. A standard curve for the *Igf1r* gene copy number was generated according to the continuous dilution of pB513B genetic recombination vector. The transgene *Igf1r* copy number per diploid cell was then calculated as described previously (Joshi et al., 2008; Wang et al., 2013). Finally, the expression of IGF-1R in MC3T3-KO was verified by FLAG antibody. The positive clones were named as MC3T3-LP and MC3T3-BM.

**TABLE 2:** Primer information for qPCR analysis of copy number.

Gene	Sequences (5′-3′)	Product lengths (bp)	Tm (°C)
PB	F: TCACGCGGTCGTTATAGTTCAA R: CCGTGAGGCGTGCTTGTC	62	58.5
β-actin	F: TTCAACACCCCAGCCATGTA R: TGTGGTACGACCAGAGGCATAC	69	58.5

### Immunofluorescence Assay

Paraformaldehyde (4%) was used to fix cells for 20 min, and 0.5% Triton X-100 in PBS was utilized to permeabilize the cells for 10 min. Subsequently, 10% fetal bovine serum was used to block the cells for 1 h. Finally, the cells were incubated with IGF-1R antibody (Abcam, United States) at 4°C overnight. On the following day, FITC-labeled immunofluorescence secondary antibody (Bioworld, United States) was added to plates of cells for incubation for 1 h in the dark. Then, 4-6-diamino-2-phenindole (Bioworld, United States) was used to visualize the nucleus. Images were captured using a fluorescence microscope (Leica, Frankfurt, Germany).

### RNA Extraction and qRT-PCR

The RNAiso Plus reagent (Takara, United States) was used to extract total RNA from the cells according to the manufacturer’s instructions. Reverse transcription was performed to generate cDNA using a reverse transcription kit. SYBR Select Master Mix (Roche) was used for Quantitative Real-time PCR (qRT-PCR). The primers used are listed in Table 3. The PCR reactions were performed on an ABI PRISM 7900HT thermocycler (Applied Biosystems, United States), and 10 ng of the cDNA template was amplified for each sample. All samples were repeated three times, and each mRNA quantification data represented the average of these three measurements. The Ct values of the target genes were normalized by the Ct value of the β-actin gene. The 2^–ΔCt^ method was employed to quantify and normalize the expression data (Cheng et al., 2020).

**TABLE 3:** Primer information for RT-qPCR analysis of expression of target genes.

Gene	Sequences (5′-3′)	Product lengths (bp)	Tm (°C)
*Col-1*	F: CCAGCCGCAAAGAGTCTACA	170	58.5
	R: TTCCACGTCTCACCATTGGG		
*Opn*	F: ACACTTTCACTCCAATCGTCC	240	58.5
	R: TGCCCTTTCCGTTGTTGTCC		
*Ocn*	F: AGACTCCGGCGCTACCTT	203	58.5
	R: CTCGTCACAAGCAGGGTTAG		
*Runx2*	F: GAGGGACTATGGCGTCAAACA	70	58.5
	R: GGATCCCAAAAGAAGCTTTGC		
*Osterix*	F: TCAGCCGCCCCGATCTTCCA	157	58.5
	R: CAATGGGTCCACCGCGCCAAG		
*Alp*	F: CAACAGGGTAGATTCTCTTGG	135	58.5
	R: GGTCAGATCCAGAATGTTCC		
*Igf1r*	F: CAAGGCTGAGAACGGCCC	160	58.5
	R: TCACTTGTCATCGTCGTCCTTG		
β*-actin*	F: GGCTGTATTCCCCTCCATCG	154	58.5
	R: CCAGTTGGTAACAATGCCATGT		

### Immunoblotting Assay

Protein content was quantified using a BCA protein assay kit (Beyotime, China) following the manufacturer’s protocols. About 40 μg of the total proteins of each sample were separated using 12 or 8% SDS-PAGE gels. The total proteins were transferred to polyvinylidene difluoride membranes. Afterward, 10% non-fat dry milk dissolved in TBST buffer was used to block the membranes for 1.5 h at 37°C. Thereafter, the primary antibodies were used to incubate with the membranes overnight at 4°C, followed by incubation with horseradish peroxidase-labeled anti-mouse IgG or anti-rabbit IgG (Bioworld, United States) for 1.5 h at room temperature. Finally, the proteins were detected using the enhanced chemiluminescence plus Western blot detection system (Amersham Biosciences). The primary antibodies used in this study were as follows: phospho-AKT (S473), AKT, and phospho-ULK-1 (S555) (Cell Signaling, Beverly, MA, United States); phosphor-mTOR (S2448), phospho-AMPK (T172 and S485), AMPK, FLAG, and IGF-1R (Abcam, United Kingdom); Beclin-1, ULK1, mTOR, OPN, and collagen I (WanLeiBio, China); and β-actin (BBI, China). Protein quantifications were analyzed using the GenoSens gel analysis software.

### Cell Proliferation

Cell proliferation assays were performed using Cell Counting Kit-8 (CCK-8, Dojindo, Japan). MC3T3-KO, MC3T3-LP, and MC3T3-BM cells were inoculated in 96-well plates at an initial density of 6.0 × 10^3^cells/well in 100 μL of culture medium.

At 0, 24, 48, and 72 h, 10 μL of the CCK-8 reagent was added to each well and reacted at 37°C for 1 h. Absorbance was detected using a microplate reader (TECAN, Switzerland) at a wavelength of 450 nm.

### Measurement of Alkaline Phosphatase (ALP) Activity

MC3T3-LP, MC3T3-BM, and MC3T3-KO cells with a density of 2.0 × 10^4^ cells/well were inoculated in 24-well plates and cultured in differentiation media. The cells were eventually harvested after 3, 5, 7, 9, 14, 18, and 21 days. Alkaline phosphatase (ALP) activities were determined using an ALP kit (Nanjing Jiancheng, China) in accordance with the manufacturer’s instructions. Protein concentrations were measured using a BCA protein assay kit (KeyGEN BioTECH, China). Enzyme activity was quantified via absorbance measurements at 520 nm using a 96-well microplate reader (TECAN, China) and calculated according to protein concentrations. Total protein content was used to normalize ALP activity, and all assays were repeated at least three times.

### Alizarin Red Staining (ARS)

Mineralization is considered the last stage of osteogenic differentiation, and the formation of mineralized nodules is a definitive marker of osteoblast mineralization. Alizarin Red (AR) chelates with calcium ions are usually used to form orange-red depositions (mineralized nodules) to determine the osteogenic mineralization ability in tissues or cells (Puchtler et al., 1969; Xi et al., 2016; Zheng et al., 2018). MC3T3-LP, MC3T3-BM, and MC3T3-KO cells with a density of 2.0 × 10^4^ cells/well were inoculated in 24-well plates and cultured in differentiation media for 7, 14, and 21 days. PBS was used to wash the cells twice before they were fixed with 70% ethanol for 1 h and stained with 1% AR (pH 7.2) for 10 min at room temperature. Afterward, the cells were washed with ddH_2_O twice and dried. The images were captured at 10 × magnification on the inverted phase-contrast microscope (Nikon, Japan) (Bae et al., 2017). The stained nodules were dissolved with 10% cetylpyridinium chloride to quantify the degree of staining, and the absorbance at 562 nm (Jiang et al., 2019) was measured using a microplate reader (TECAN, China). The results were replicated in at least three independent experiments.

### Stability Assay

MC3T3-LP and MC3T3-BM cells with a density of 3.0 × 10^5^ cells/well were inoculated in 6-well plates. Actinomycin D (ActD, 5 μg/mL) and cycloheximide (CHX, 50 μg/mL) were added to the medium and used to block mRNA transcription and protein synthesis respectively. The cells were collected at 0, 1, 2, 3, and 4 h after culture, and the total RNAs and proteins were extracted. The stability of the mRNAs and proteins was detected from the collected RNAs and proteins, respectively.

### Co-immunoprecipitation Analysis (Co-IP)

Exactly 500 nM IGF-1 was added to the medium because previous studies reported that 500 nM IGF-1 reaches the maximum binding concentration when IGF-1R is combined with IGF-1 (Jansson et al., 1997; Whitten et al., 2009). The MC3T3-LP, MC3T3-BM and MC3T3-KO cells were incubated in the medium containing IGF-1 for 6 h. Subsequently, the cells were collected and lysed. Co-IP incubation was performed using Pierce^TM^ protein A/G magnetic beads (Thermo, United States). The lysate was centrifuged at 13,000 × *g* for 15 min, and the supernatant was immunoprecipitated overnight with IGF-1R antibody or IgG antibody (control). On the following day, the magnetic beads and the antigen-antibody complex was incubated for 2 h at 4°C. The collected protein and magnetic bead complexes were washed five times with a wash buffer and eluted with an elution buffer. Finally, the eluents were neutralized with a neutralization buffer and boiled in a protein sample buffer under reducing conditions. The sample proteins were resolved and analyzed via SDS-PAGE analysis and Western blot, respectively.

### Flow Cytometry Analysis

The MC3T3-LP, MC3T3-BM and MC3T3-KO cells were incubated with 4% paraformaldehyde alone or incubated with 4% paraformaldehyde and permeabilized with 0.1% Triton X100 to evaluate the cell surface expression of IGF-1R in the cells (Rezgui et al., 2009). The cells were stained with FITC-conjugated anti-FLAG antibody (1:400, Abcam, United States) and analyzed via flow cytometry (BD FACSAriaII) (Ip et al., 2018).

The MC3T3-LP, MC3T3-BM and MC3T3-KO cells were incubated in 4% paraformaldehyde for 20 min and permeabilized with 0.5% Triton X-100 in PBS for 10 min for IGF-1R staining to evaluate the differences in protein conformations of IGF-1R. Subsequently, 5% goat serum was used to block the cells for 1 h, and the primary antibody against IGF-1R (1:80, Abcam, United States) was used to incubate the cells overnight at 4°C. On the following day, an FITC-labeled secondary antibody (1:100, Bioworld, United States) was used to incubate the cells for 1 h in the dark. The fluorescence intensity of a population of 10,000 cells was measured with the fluorescence associated with the FITC. IgG-treated cells were used as negative controls (Fung et al., 2014).

Subsequently, 5% goat serum was used to block the cells for 1 h, and the primary antibody against IGF-1R (1:80, Abcam, United States) was used to incubate the cells overnight at 4°C. On the following day, an FITC-labeled secondary antibody (1:100, Bioworld, United States) was used to incubate the cells for 1 h in the dark. The fluorescence intensity of a population of 10,000 cells was measured with the fluorescence associated with FITC. IgG-treated cells were used as negative controls.

### Statistics Analysis

All experimental results are presented as the mean ± SEM of at least three independent experiments. One-way or two-way ANOVA was used to test statistical differences among groups. All statistical analyses were performed using GraphPad Prism 6.0. Differences were considered significant at *p* < 0.05 (*^∗^p* < 0.05, *^∗∗^p* < 0.01).

## Results

### Generation of *Igf1r*-Knockout MC3T3-E1 Clones

Four homozygous mutations (c.931G > C, c.1507T > G, c.1762C > T, and c.2269 T > C) in the CDS of IGF-1R ECD from pigs of different body size traits were all synonymous mutations (Figure 1A). Two haplotypes, of which GTCT is the haplotype of BM pigs and CGTC is that of large pigs, were formed (Figure 1B).

To investigate the role of different *Igf1r* haplotypes in bone development and avoid endogenous *Igf1r* interference in osteoblasts, we first constructed *Igf1r*-knockout in MC3T3-E1 cells by using the CRISPR/cas9 system. To ensure complete *Igf1r* knockout, we designed one target site on the exon 4 of *Igf1r* sequences to target the common sequences contained in all the alternative splicing isoforms. The position of the target site was relatively close to the beginning of the sequence (Figure 1C). sgRNA sequences were then connected to plasmid PX458 and transfected into MC3T3-E1 cells. Fluorescence-activated cell sorting was implemented to select the cells expressing GFP fluorescence to improve the efficiency of positive cloning screening. The presence of IGF-1R mutations in genomic DNA was identified via DNA sequencing. Most cell clones showed frameshift mutation, and one cell clone had a nucleotide deletion near the protospacer adjacent motif (Figure 1C). To assess knockout efficiency, we performed immunofluorescence staining on the *Igf1r*^Knockout^ cell clones by using the IGF-1R antibody. The MC3T3-E1 cells exhibited strong IGF-1R signals, whereas the *Igf1r*^Knockout^ cell clones lost the IGF-1R signal (Figure 1D). Meanwhile, the IGF-1R protein levels in different mutant clones were measured via immunoblotting. The *Igf1r*^Knockout^ cell clones lost the IGF-1R protein expression as indicated by the Western blot results (Figures 1E,F). We selected a clone with sufficient *Igf1r* knockout and named MC3T3-knockout cell.

### Stable Expression of Two Haplotypes of *Igf1r* in MC3T3-KO Cells

The pB513B-LP and pB513B-BM vectors were co-transfected with the PB transposase expression plasmid into MC3T3-KO cells, respectively, and normal MC3T3-KO cells were used as a control. The co-transfected cells were selected by puromycin for 10 days to obtain puromycin-resistant cell clones. About 50 cell clones per well were left after the co-transfection of the screened PB dual vector system. Almost no cell clones survived in the control group, indicating that the cell clones that survived puromycin selection were positively transposed cell clones.

**FIGURE 2:**
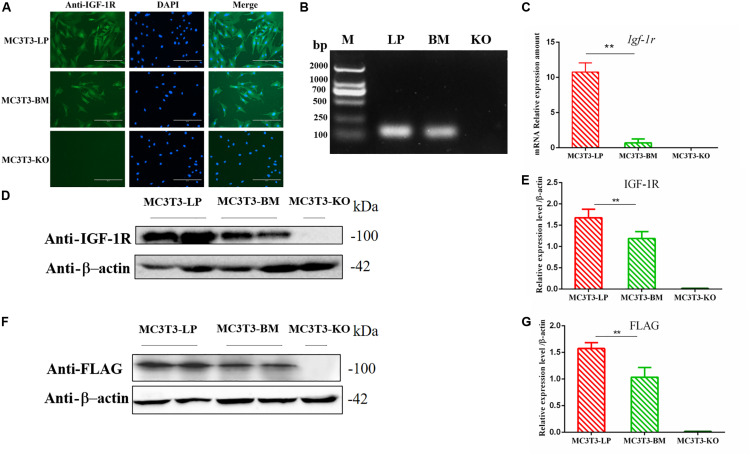
Generation of recombinant cell lines of two haplotypes of *Igf1r* and effect of the haplotypes of *Igf1r* on gene transcription, translation. **(A)** Immunostaining of IGF-1R (Green) and DAPI (Cyan) in MC3T3-LP and MC3T3-BM cells. **(B)** PCR results of the MC3T3-LP (LP), MC3T3-BM(BM) and MC3T3-KO (KO)cells. **(C)** RT-qPCR analysis of IGF-1R expression in MC3T3-LP, MC3T3-BM and MC3T3-KO cells. **(D**,**F)** Western blot analysis of the IGF-1R and FLAG expression in MC3T3-LP, MC3T3-BM and MC3T3-KO cells and identified with anti-IGF-1R and anti-FLAG antibodies, respectively. **(E**,**G)** Quantification of the **(D,F)** western blot results, respectively (**p < 0.01).

Genomic DNA was extracted from transposed MC3T3-KO cells and MC3T3-KO control cells to evaluate whether exogenous genes had been stably integrated into the genome. The expression level of exogenous genes was then detected. Specific PCR products corresponding to the two haplotypes of *Igf1r* sequences were observed from the transposed cells, whereas no PCR products were found from the control cells without transposed cells. qPCR was performed to detect the two integrated haplotypes of *Igf1r* gene copy number and analyze 10 clonal cell lines for each haplotype. The cell clones had 3–13 copies with an average of five copies per clone. We finally selected two cell clones with haplotypes of five copies each. The copy number of our clones was similar to that reported by previous studies in which mammalian cells were transfected with the PB transposon system (Ding et al., 2005; Grabundzija et al., 2010). Finally, puromycin-resistant cell clones with two different strong IGF-1R signals were observed via immunofluorescence staining (Figure 2A). These cell clones were named as MC3T3-LP cells (LP group) and MC3T3-BM cells (BM group).

### Effects of Two Haplotypes of *Igf1r* on the IGF-1R mRNA and Protein Expression Level

The expression levels of IGF-1R in MC3T3-LP cells containing the CGTC haplotype and MC3T3-BM cells containing the GTCT haplotype were detected to clarify the biological effects of these haplotypes. A reverse primer was designed on the FLAG tag sequences to avoid the interference of intracellular IGF-1R in detecting the mRNA expression level (Figure 2B). The mRNA expression levels were significantly lower in the BM group than in the LP group (Figure 2C) (*P* < 0.01). The protein expression level of each group was detected using an anti-IGF-1R antibody (Figures 2D,E) (*P* < 0.01) and anti-FLAG tag antibody (Figures 2F,G) (*P* < 0.01). Results consistently showed that the mRNA and protein expression levels of the BM group were lower than those of the LP group. Given that changes in expression levels can affect the biological effects of genes, we speculated that different haplotypes of *Igf1r* may also affect its function based on the observed differences in expression levels.

### Effect of Two Haplotypes of *Igf1r* on Cell Proliferation

**FIGURE 3:**
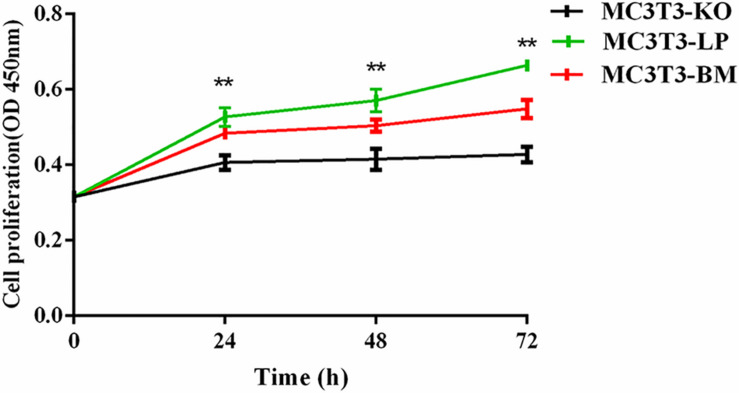
Effect of two haplotypes of *Igf1r* on cell proliferation in MC3T3-LP, MC3T3-BM and MC3T3-KO cells, CCK-8 proliferation assay of MC3T3-LP, MC3T3-BM and MC3T3-KO cells after starved in DMEM containing 3% FBS for 3 h at 0, 24, 48, and 72 h (**P < 0.01).

To verify the functions of IGF-1R ECD encoded by the haplotypes in Bama Xiang and large pigs, we investigated the cell proliferation abilities of MC3T3-LP and MC3T3-BM cells and compared them with that of MC3T3-KO cells. CCK-8 assay revealed that the cell proliferation ability of the LP group was more substantial than that of the BM and KO groups from 0 to 72 h (Figure 3) (*p* < 0.01), suggesting that the cells of the BM group had a lower growth potential than the cells of the LP groups.

### Effect of Two Haplotypes of *Igf1r* on the ALP Activity of Osteoblasts

**FIGURE 4:**
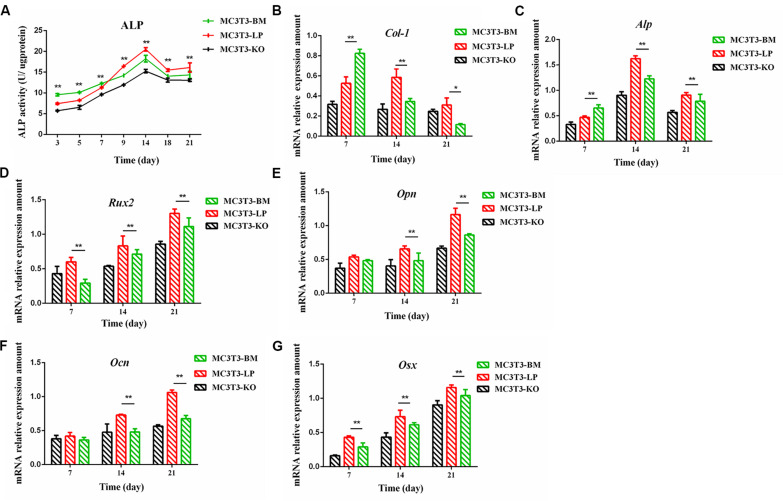
Effect of two haplotypes of *Igf1r* on ALP activity and the mRNA amount of differentiation related gene in MC3T3-LP, MC3T3-BM and MC3T3-KO cells. **(A)** ALP activity assay of MC3T3-LP, MC3T3-BM and MC3T3-KO cells on days 7, 14 and 21, respectively. **(B–G)** RT-qPCR analysis of *Col-1*, *ALP*, *Runx2, Opn*, *Ocn* and *Osx* expression levels in MC3T3-LP, MC3T3-BM and MC3T3-KO cells on days 7, 14, and 21, respectively (**P < 0.01).

The proliferation and differentiation of osteoblasts are essential factors in bone growth. On the basis of our observations of the effects of cell proliferation, we further detected the effects of different haplotypes of *Igf1r* on osteoblast differentiation. ALP activity is a marker of early osteogenic differentiation that is used to evaluate the effects of different *Igf1r* haplotypes on cell differentiation (Golub et al., 1992; Bancroft et al., 2002; Hosseini et al., 2019). The MC3T3-LP, MC3T3-BM, and MC3T3-KO cells were maintained in a differentiation medium. The ALP activities of the LP, BM, and KO groups were separately measured on days 3, 5, 7, 9, 14, and 21. As shown in Figure 4A, the ALP activities of all three groups fluctuated: the trend initially fell and then rose from day 3 to day 21. Meanwhile, the ALP activity of the BM group was significantly higher than that of the LP and KO groups from day 3 to day 7 (*P* < 0.01). Thereafter, the ALP activity of the LP group was significantly higher than that of the MC3T3-BM and MC3T3-KO groups (*P* < 0.01). Compared with the LP group, the BM group promoted ALP activity at the early stage of differentiation but significantly inhibited ALP activity at the late stage of differentiation. However, the detection of ALP activity alone was insufficient to prove that the different haplotypes of *Igf1r* affected osteoblast differentiation as this process is regulated by numerous genes and transcription factors. We speculated that the haplotypes of *Igf1r* may affect the expression of genes related to bone differentiation.

### Effect of the Two Haplotypes of *Igf1r* on the Expression of Genes Related to Osteogenic Differentiation

We explored how the genotypes of *Igf1r* affect osteogenic differentiation. We investigated the expression of the genes related to osteogenic differentiation in the BM, LP, and KO groups. The expression levels of collagen-1 (*Col-1*), osteocalcin (*Ocn*), osterix (*Osx*), osteopontin (*Opn*), runt-related transcription factor 2 (*Runx2*), and *Alp* were significantly different in the cells carrying the two haplotypes of *Igf1r*. On the 7th day of differentiation, the mRNA expression level of *Col-1* and ALP was lower in the KO and LP groups than that in the LP group (Figures 4B,C) (*P* < 0.01). However, the mRNA expression level of *Opn*, *Ocn*, *Runx2*, and *Osx* were higher in the LP group than that in the KO and BM groups on days 7, 14, and 21 (Figures 4D–G) (*P* < 0.01).

**FIGURE 5:**
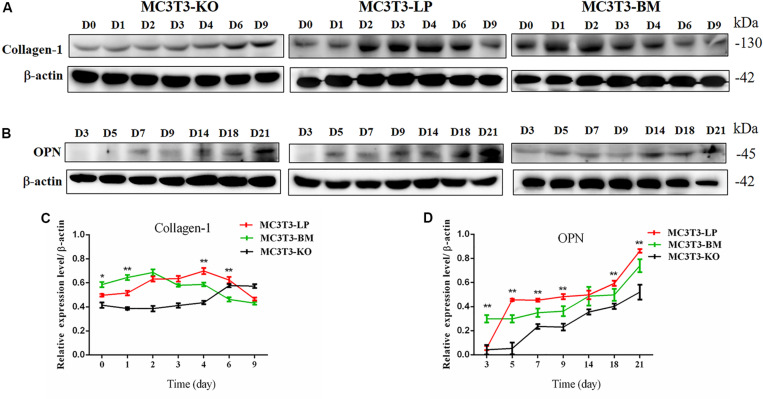
Effect of two haplotypes of *Igf1r* on the protein amount Col-1 and OPN. **(A)** Western blot analysis of Col-1 expression in MC3T3-LP, MC3T3-BM and MC3T3-KO cells on days 0, 1, 2, 3, 4, 6, and 9. **(B)** Western blot analysis of OPN expression in MC3T3-LP, MC3T3-BM and MC3T3-KO cells on days 3, 5, 7, 9, 14, 18 and 21. **(C)** The line chart of the **(A)** after a gray scale analysis. **(D)** The line chart of the **(B)** after a gray scale analysis (*P < 0.05, **P < 0.01).

*Col-1* induces extracellular matrix formation at the early stages of osteocyte differentiation, whereas *Opn* acts as a mediator of mineralization (Chai et al., 2019; Hosseini et al., 2019). The expression levels of these two genes play a crucial role in osteoblast differentiation. Western blot revealed that the expression level of Col-1 was higher in the BM group than that in the LP group on days 1 and 2 but lower from days 3 to 6 (*P* < 0.01) (Figures 5A,C). Furthermore, the protein expression level of OPN was markedly attenuated in the BM group compared with that in the LP group from days 3 to 21 (Figures 5B,D) (*P* < 0.01). These results were consistent with the results described in the preceding section.

### Effects of the Two Haplotypes of *Igf1r* on Mineralized Nodules

**FIGURE 6:**
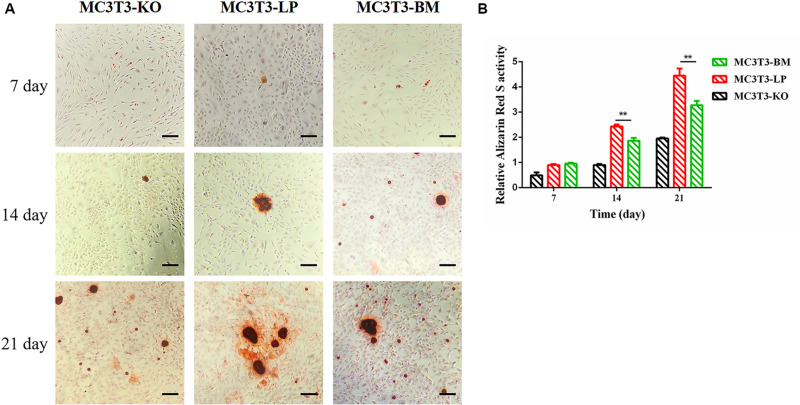
Effect of two haplotypes of *Igf1r* on cell mineralization in MC3T3-LP, MC3T3-BM and MC3T3-KO cells. **(A)** Mineralized nodules of MC3T3-LP, MC3T3-BM and MC3T3-KO cells on days 7, 14, and 21. The images were captured on the inverted phase microscope (scale bar, 200 μm). **(B)** The quantified result of mineralization by 10% cetylpyridinium chloride (**P < 0.01).

Calcium deposition is an important indicator of matrix mineralization in bone differentiation. AR, which preferentially chelates with calcium ions, is widely used to identify matrix mineralization (Puchtler et al., 1969). A previous study indicated that the mineralization of MC3T3-E1 cells occurs in a time-dependent manner and can be examined during osteoblast differentiation for up to 21 days (Xi et al., 2016). The initial formation of mineralized nodules was observed on day 7. The mineralized nodules gradually increased with the extension of culture time and degree of differentiation. After induction of osteoblast mineralization, ARS revealed that the BM group had slightly less mineralized nodules compared with the LP and KO groups on day 7. The modules were remarkably larger in the LP group than those in the BM and KO groups on days 14 and 21 (Figures 6A,B) (*P* < 0.01). The results suggested that the BM group promoted the early stage of osteogenic mineralization, whereas the LP group promoted the late stage of osteogenic mineralization. Both groups exhibited similar trend in terms of the expression levels of genes related to osteogenic differentiation. On the basis of osteogenic differentiation, we observed that the regulation of the haplotypes of *Igf1r* were different at various stages of differentiation. We speculated that the haplotypes of *Igf1r* may affect the expression of genes related to bone development by affecting the signaling pathways associated with intracellular osteogenic differentiation.

### Effects of the Two Haplotypes of *Igf1r* on the Signal Output of Osteogenic Differentiation

**FIGURE 7:**
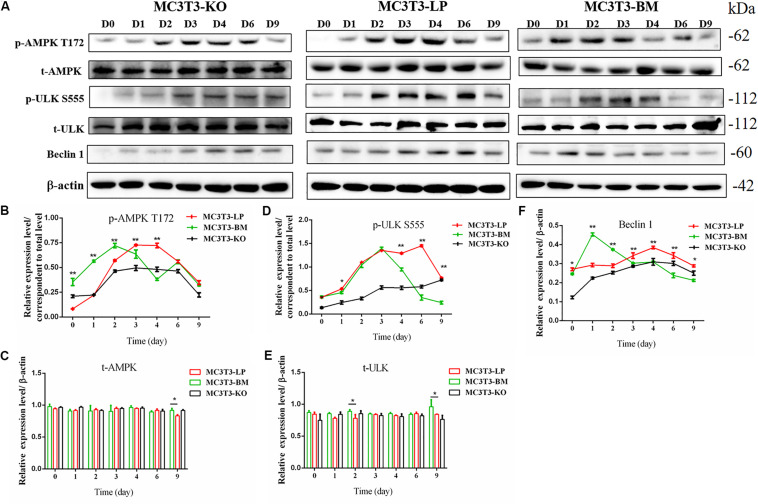
Effect of two haplotypes of *Igf1r* on the signal output of early-stage osteogenic differentiation. **(A)** Western blot evaluation results of AMPK-T172, T-AMPK, ULKS55, T-ULK, and Beclin-1 protein expression in MC3T3-LP, MC3T3-BM and MC3T3-KO cells, respectively on days 0, 1, 2, 3, 4, 6, and 9. **(B–F)** Quantification of the **(A)** western blot results (*P < 0.05, **P < 0.01).

**FIGURE 8:**
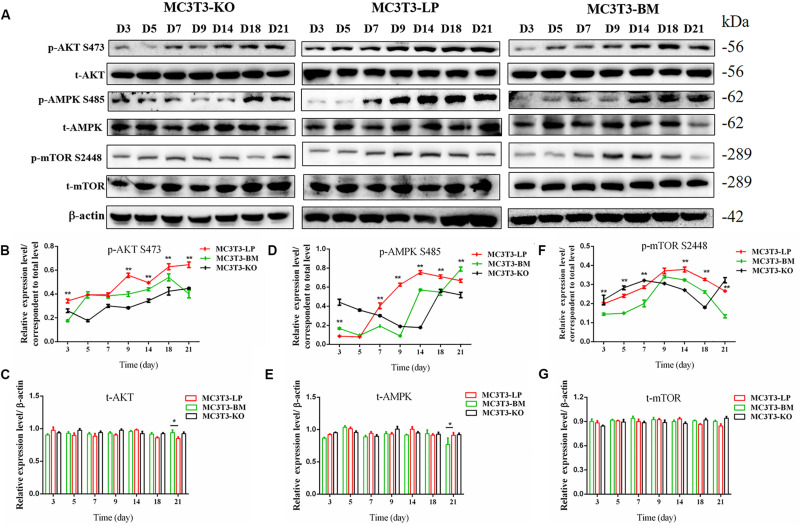
Effects of two haplotypes of *Igf1r* on the signal output of late stage of differentiation. **(A)** Western blot evaluation results of P-AKT (473), T-AKT, P-AMPK S485, T-AMPK, mTOR S 2448 and mTOR protein expression in MC3T3-LP, MC3T3-BM and MC3T3-KO cells, respectively, on days 3, 5, 7, 9, 14, 18, and 21. **(B–G)** Quantification of the **(A)** western blot results (*P < 0.05, **P < 0.01).

Induction of autophagy is required in early differentiation, and the binding of IGF-IR with IGF-1 can activate different AMPK phosphorylation sites at different stages of cell differentiation to regulate autophagy stimulation or inhibition (Xi et al., 2016). Given that phosphorylation of AMPK-T172 directly phosphorylates unc-51-like autophagy-activating kinase-1 S555 (ULK-1 S555), we compared the phosphorylation of AMPK-T172 and ULK-1 S555 and the expression level of Beclin-1 (another critical component of autophagosomes) at the early stage of differentiation. Given that the late activation of mTOR S2448/AKT S473/AMPK S485 occurs in the osteogenic differentiation of human mesenchymal stem cells and MC3T3-E1 cells (Pantovic et al., 2013; Xi et al., 2016), we then detected the phosphorylation levels of mTOR S2448, AKT S473, and AMPK S485 at the late stage of differentiation. AMPK-T172 phosphorylation level and Beclin-1 expression level initially increased in the BM group from day 0 to 2, but the phosphorylation level of ULK-1 S555 was not significantly different. Moreover, Beclin-1 expression level and AMPK-T172 and ULK-1 S555 phosphorylation levels evidently decreased in the BM group compared with those in the LP group from day 3 to 9 (Figure 7). However, at the relatively late stage of differentiation, mTOR S2448, AKT S473, and AMPK S485 phosphorylation levels in the BM group continued to decrease from day 3 to 21 (Figure 8).

These results suggested that the two haplotypes of *Igf1r* play different roles in regulating the downstream signaling pathway of osteoblast differentiation. The osteoblasts in the BM group had a higher AMPK phosphorylation level and Beclin-1 expression level compared with the LP group at the early stage of differentiation, and the osteoblasts inhibited autophagy from the third day of differentiation.

### Effects of the Two Haplotypes of *Igf1r* on mRNA and Protein Stability

**FIGURE 9:**
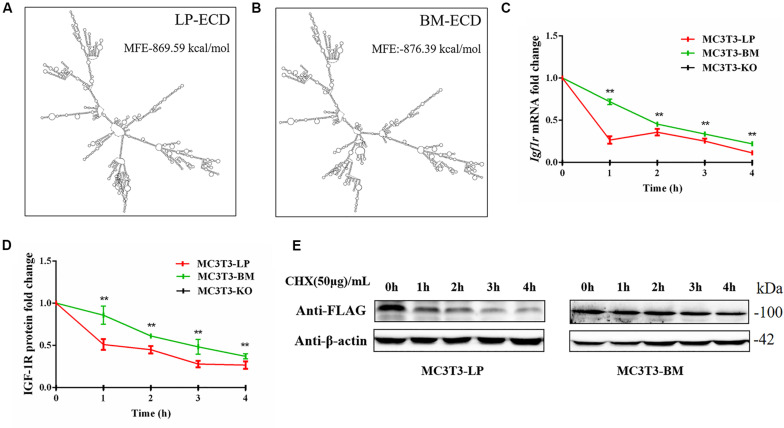
The effect of two haplotypes of *Igf1r* on the stability of mRNA and protein **(A, B)** mRNA secondary structure and minimum free energy of two haplotypes of *Igf1r* ECD cDNA sequence from large pig and Bama Xiang pig. **(C)** MC3T3-LP and MC3T3-BM cells were treated with ActD for 0–4 h. The level of IGF-1R was analyzed. **(D)** MC3T3-LP and MC3T3-BM cells were treated CHX for 0–4 h. The level of IGF-1R was analyzed by western blot and β-actin was used as a loading control. **(E)** Line chart of the **(D)** after the gray scale analysis (**P < 0.01).

Synonymous mutations can affect gene expressions by affecting gene stability and translation folding (Hunt et al., 2014; Cheng et al., 2018). We speculated that the haplotypes of *Igf1r* may be influenced by differences in mRNA and protein stability. On the basis of the differences in the expression level of *Igf1r* in the BM and LP groups at the transcription and translation levels (Figures 2D,E,G), we evaluated the differences in mRNA and protein structural stabilities between the two haplotypes of *Igf1r*. The secondary mRNA structures and minimum free energies of the two haplotypes of *Igf1r* ECD were predicted through the bioinformatics website RNAfold web server. Results showed that the secondary mRNA structures of the two haplotypes of *Igf1r* ECD were different, and the minimum free energy increased by 6.8 kcal when the GTCT haplotype carried by the BM group was converted to the CGTC haplotype carried by the LP group (Figures 9A,B). In combination with these results and the differences in the expression levels of the two haplotypes of *Igf1r* previously detected, we speculated that these haplotypes may regulate gene expression levels on the basis of theory of codon translation efficiency. In other words, the translation rates of individual codons might vary by 5- to 25-fold (Curran and Yarus, 1989; Srensen and Pedersen, 1991). Another study reported that stabilization by a mutation of the initiator helix by -3.6 kcal/mol resulted a 500-fold drop in expression (Smit and Duin, 1990).

We examined the mRNA stability of the two haplotypes. The MC3T3-LP and MC3T3-BM cells were treated with the mRNA transcription inhibitor ActD, respectively. Results showed that the mRNA stability of *Igf1r* in the MC3T3-BM cells was higher than that in the MC3T3-LP cells (*P* < 0.01) (Figure 9C). This result was consistent with the prediction outcomes of mRNA secondary structures. Meanwhile, the protein stability test results showed that the IGF-1R protein degradation in MC3T3-BM cells was more slowly than that in MC3T3-LP cells (*P* < 0.01) (Figures 9D,E).

### Different *Igf1r* Haplotypes Alter Binding Affinity With IGF-1

**FIGURE 10:**
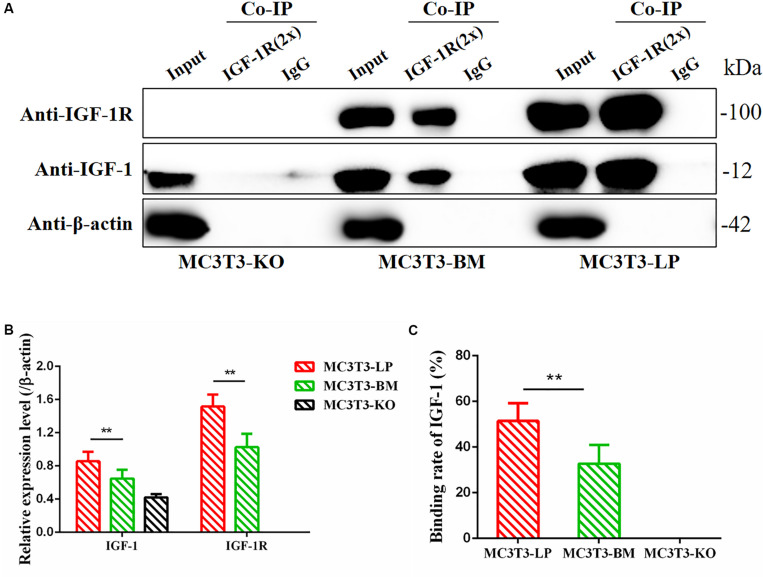
Binding affinity analysis of the IGF-1 with IGF-1R in MC3T3-KO, MC3T3-BM and MC3T3-LP cells **(A)** MC3T3-KO cells (the KO group), MC3T3-BM cells (GTCT haplotype of BM group) and MC3T3-LP cells (CGTC haplotype of LP group) were lysed, IGF-1R was immunoprecipitated and IgG served as a negative control, IGF-1 and IGF-1R were then examined by western blot. **(B)** Quantification of IGF-1 and IGF-1R levels in the immunoprecipitated complex. **(C)** Quantification of the related binding rate with IGF-1 (**P < 0.01).

Considering that the four synonymous mutations on IGF-1R ECD are located near the binding site with the ligand IGF-1, we detected the interactions between IGF-1 and IGF-1R in MC3T3-LP, MC3T3-BM, and MC3T3-KO cells via co-immunoprecipitation assay. As shown in Figure 10, except that IGF1R was not expressed in MC3T3KO cells, different amounts of IGF-1 and IGF-1R were the inputs of MC3T3-LP, MC3T3-BM and MC3T3-KO cells (Figures 10A,B) (*P* < 0.01). Additionally, IGF-1 was co-immunoprecipitated with IGF-1R when it was immunoprecipitated by the IGF-1R antibody (Figure 10A). No binding was detected between IGF-1 and IGF-1R in MC3T3-KO cells, excluding the interference of non-specific binding, and compared with IGF-1 in the input, the amount of IGF-1 that bound with IGF-1R in the MC3T3-LP cells was higher than that in the MC3T3-BM cells (Figure 10C) (*P* < 0.01). The difference in the binding rate between IGF-1R and IGF-1 can be influenced by several factors, such as the expression of IGF-1R on cell surfaces and the conformation of IGF-1R proteins. Thus, we detected the expression level of IGF-1R on the surface of cell membranes and the conformation of IGF-1R proteins.

### Effect of the Two Haplotypes of *Igf1r* on Membrane Surface Expression and Protein Conformation

**FIGURE 11:**
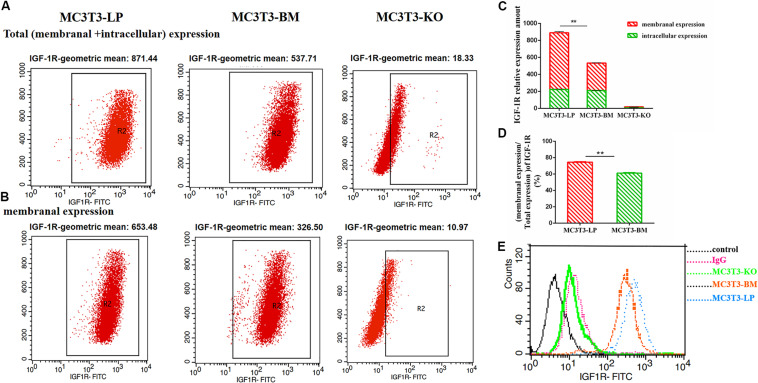
The effect of two haplotypes of *Igf1r* on membrane bound expression and conformation of IGF-1R. **(A,B)** Flow cytometry of IGF-1R membrane bound expression with anti-FLAG antibody in MC3T3-LP, MC3T3-BM and MC3T3-KO cells. **(C)** Quantification of IGF-1R membranal expression and intracellular expression levels in MC3T3-LP, MC3T3-BM and MC3T3-KO cells. **(D)** Quantification of the related membranal expression rate in MC3T3-LP, MC3T3-BM cells (**P < 0.01). **(E)** Flow cytometry assay to detect the conformation of IGF-1R of MC3T3-BM cells (GTCT haplotype of BM group, yellow line), MC3T3-LP cells (CGTC haplotype of LP group, blue line) and MC3T3-KO cells (green line).

To determine the reasons for the decrease in affinity of the two haplotypes of *Igf1r* with IGF-1, we detected IGF-1R distributions across the MC3T3-LP, MC3T3-BM and MC3T3-KO cell membranes by using the anti-FLAG antibody. The expression of IGF-1R on cell membrane surface in fixed and non-permeabilized cells and the expression of total IGF-1R in fixed and permeabilized cells were quantitatively detected via flow cytometry. Almost no fluorescence was detected in MC3T3-KO cells. Excluding the interference of non-specific binding, the total protein and IGF-1R expression levels on the surfaces of cell membranes were lower in the BM group than those in the LP group (Figures 11A–C) (*p* < 0.01). Moreover, the relative membranal expression level of IGF-1R was lower in the BM group than that in the LP group (Figure 11D) (*p* < 0.01).

Monitoring the antigen-antibody interaction of a protein in an intact cell by using flow cytometry has the considerable advantage of allowing a conformation-sensitive antibody to explore the tertiary structure of a functional protein in its native environment (Sauna et al., 2009). To determine whether the four synonymous mutations of *Igf1r* in ECD changed their protein conformations, we incubated the MC3T3-LP and MC3T3-BM and MC3T3-KO cells with the IGF-1R antibody, the KO group as a negative control group. We detected the fluorescence intensity of each haplotype by using a flow cytometer to reflect the binding rate between IGF-1R and its antibody. The distribution of fluorescence intensity in a population of 10,000 cells is shown in Figure 10E. The cells treated with the IgG antibody established a baseline with a low level of fluorescence that intuitively represented non-specific interactions and interactions with the secondary antibody alone. The fluorescence intensity of the binding of MC3T3-KO cells to the antibody was similar to that of IgG antibody, which could exclude the endogenous interference of MC3T3-KO cells. In addition, the MC3T3-LP and MC3T3-BM cells exhibited specific binding to the *Igf1r* antibody. The MC3T3-LP cells showed a higher increase in fluorescence than the MC3T3-BM cells, indicating that the *Igf1r* haplotypes of large pigs have a more remarkable affinity with the antibody than the *Igf1r* haplotypes of Bama Xiang pigs. Thus, the two haplotypes of *Igf1r* generated by the four synonymous mutations affected the membrane surface expression and protein conformation of IGF-1R.

## Discussion

Miniature pigs have likely become important laboratory animals and xenotransplantation donors in biomedical research (Prather et al., 2013). However, the formation of miniature pigs remains unclear. IGF-1R is composed of seven extracellular domains, namely, L1, CR, L2, Fn1, Fn2, ID, and Fn3, as well as a transmembrane (TM) region and an intracellular domain (Baxter, 2015). The L1, ID, and Fn3 domains are the key regions where IGF-1R binds with IGF-1 (Adams et al., 2000). A previous study demonstrated that the five synonymous mutations of IGF-1R ICD affect cell proliferation and alter kinase activity (Wang et al., 2020). By contrast, in the present study, we discussed the four synonymous mutations identified in the IGF-1R ECD of large and miniature pigs. These mutations were located at the CR, Fn1, and ID structural domains of IGF-1R, as well as near the IGF-1 binding domains. IGF-1R, activated by IGF-1, mediates a conserved signaling pathway necessary for stimulating osteoblast proliferation (LeRoith et al., 1995), thereby accelerating their differentiation and enhancing bone matrix production (Xi et al., 2016). Loss of IGF-1R leads to severe growth deficiency and developmental delays in ossification (Baker et al., 1993). Mice with targeted IGF-1R overexpression in osteoblasts exhibit an increased bone formation rate and an enhanced trabecular and cortical bone volume (Zhao et al., 2000). Numerous studies demonstrated that synonymous mutations have considerable biological effects on various processes, such as gene expression, gene stability, and protein conformation (Fung et al., 2014; Chaney and Clark, 2015; Cheng et al., 2018). As a ligand-dependent receptor, stability and conformational changes in IGF-1R regulate IGF-1R activity and lead to differences in downstream functions. Moreover, previous studies corroborated the relationship between gene SNPs and bone phenotypes in animals (Horvath et al., 2016; Bin et al., 2017; Gaffney-Stomberg et al., 2017). Therefore, we suspected that these synonymous mutations of porcine IGF-1R ECD most likely play a vital role in regulating IGF-1R expression, as well as in the regulation of the proliferation, differentiation, and mineralization of osteoblasts.

We first investigated the effects of two *Igf1r* haplotypes formed by four linkage synonymous mutations on gene expression. The results were consistent with our expectations, indicating that the expression levels in the BM group were lower than those in the LP group at both the transcription and translation levels. We observed different effects of the two *Igf1r* haplotypes on promoting cell proliferation. The KO group had the lowest proliferation capacity, thus indirectly indicating that IGF-1R plays an important role in cell proliferation. Furthermore, the LP group had a better effect on promoting cell proliferation than the BM group. These results suggested that the preferred codons in large pigs are more conducive to cell proliferation and survival. Moreover, the four synonymous mutations of porcine *Igf1r* ECD may contribute to the formation of body size in animals.

We also found that IGF-1R is involved in the regulation of osteoblast differentiation and mineralization. Among the indicators of cell differentiation, ALP activity is considered one of the most essential factors affecting osteoblast differentiation (Whyte, 1994). ALP generates calcium phosphate to be deposited in mature osteoblasts and contributes to bone mineralization (Orimo and Shimada, 2008; Hosseini et al., 2019). Aside from ALP activity, the expression levels of genes related to osteogenic differentiation (*Col-1*, *Alp*, *Opn*, *Ocn*, *Rux2*, and *Osx*) were also found to be important factors for evaluating cell differentiation. *Col-1* and *Alp* induce the formation of the primary extracellular matrix (ECM) at the early stage of differentiation (Hosseini et al., 2019). The early differentiation of pre-osteoblasts is regulated by *Runx2* (Ducy et al., 1997). *Runx2* and *Osx* play a regulative role in regulating osteoblast differentiation and bone formation in growing bones (Baek et al., 2009). *Ocn* is a marker at the late stage of osteoblast differentiation (Hosseini et al., 2019). *Opn* is a highly phosphorylated glycoprotein secreted during osteoblast differentiation and differentiation, the expression level of which gradually increases from days 7 to 21 (Letzring et al., 2010; Wei et al., 2020). Hence, *Opn* can be used as an indicator of the intermediate and late stages of differentiation. These genes play an essential role in regulating osteoblast differentiation and bone formation in growing bones. A previous study reported that the expression of *Col-1* increases at the early stage and then decreases at the later stage of differentiation (Hosseini et al., 2019; Wei et al., 2020). The expression level of *Opn* continues to increase on the 7th day of differentiation (Wei et al., 2020). On the basis of these reports, we selected *Col-1* and *Opn* as the indicators of early and late differentiation stages, respectively, for follow-up studies.

The ALP activity of the LP and BM groups was substantially higher than that of the KO. The BM group promoted the early stage of osteogenic differentiation, whereas the LP group promoted the late stage of osteogenic differentiation. This observation was further confirmed by the detection of the expression levels of genes related to osteogenic differentiation. Matrix mineralization is the final step in osteoblast differentiation. It marks osteogenic maturation ability and plays a crucial role in regulating bone size (i.e., bone mass and volume). AR stains are widely used to detect mineral deposition (Puchtler et al., 1969). In this study, the number of mineralized nodules was substantially greater in the LP and BMs than in the KO group. Moreover, the nodules in the BM group decreased compared with in the LP group. This trend was consistent with ALP activity. Similar to the findings of previous studies, the present results confirmed that IGF-1R regulates osteoblast differentiation and mineralization. Furthermore, codon preference plays a role in regulating gene functions to a certain extent.

Like most receptors, IGF-1R is involved in or cross-talk with various signal pathways (Larsson et al., 2005; Tandon et al., 2016). Autophagy is a phenomenon that cannot be ignored during cell differentiation. It is regulated by multiple cellular pathways. Stimulation of autophagy is necessary for early osteoblast differentiation, which requires biphasic regulation of AMPK. IGF-1R signaling stimulates AMPK activation, thereby resulting in autophagy activation at the early stage of differentiation (Xi et al., 2016). Alternatively, AKT activation stimulates AMPK S485 phosphorylation (Sarbassov et al., 2005), thus leading to the suppression of AMPK activation (reduction of T172 phosphorylation) and the termination of autophagy between days 9 and 15 (Xi et al., 2016). Therefore, we detected the related downstream signaling pathways of IGF-1R via Western blot. In contrast to the LP group, the BM group first upregulated the phosphorylation of AMPK and the expression of autophagosome components at the early stage of osteoblast differentiation. Thereafter, the LP group remained suppressed at the late stage of differentiation. These results suggested that the two haplotypes of IGF-1R may affect their differentiation patterns by changing the phosphorylation levels of downstream pathways during the period of AMPK induction. We further indicated that the haplotype of the BM group can promote early stage differentiation and delay the late stage differentiation. Furthermore, the haplotype of the BM group inhibits the expression of bone-related genes (*Rux2*, *Alp*, *Ocn*, *Opn*, and *Osx*) and decreases ALP activity and calcium deposition. Prior studies confirmed the positive selection of early maturation in the development of Bama Xiang pigs. Functional enrichment analysis indicated that autophagy regulation is one of the most important biological processes (Yang et al., 2018). From day 0 to day 160, the growth curve of miniature pigs is approximately linear. By contrast, normal, fattening pigs have low average daily gain during the early growth stage, and it gradually increases after day 50 (Kohn et al., 2007). The results of previous studies were consistent with our findings that osteoblast differentiation in miniature pigs is more active during the early stage of growth and development than during the other growth stages. Therefore, we speculated that the polymorphisms of IGF-1R play a specific role in regulating the unique growth characteristics of miniature pigs. Further research is warranted to verify this hypothesis.

mRNA and protein stabilities can be affected by a synonymous codon change (Pedersen, 1984; Letzring et al., 2010). Chaney et al. demonstrated that the temporal coordination of co-translational folding is an additional mechanism of synonymous mutation in regulating translation dynamics (Chaney and Clark, 2015). Moreover, stable mRNA structures can lead to translational pauses (Tu et al., 1992). Thus, the issue of whether the difference in functions between two haplotypes is due to stability changes remains uncertain. In this study, a significant difference was predicted between the mRNA secondary structures of the two different sequences of IGF-1R ECD. When the GTCT haplotype of the BM group changed to the CGTC haplotype of the LP group, the minimum free energy increased by 6.8 kcal. In addition, the mRNA stability of IGF-1R with the BM haplotype was higher than that of the LP haplotype, which was consisted with the prediction result of mRNA secondary structures and the minimum free energy for two haplotypes. A number of studies have shown that protein synthesis is affected by many factors, such as mRNA expression level, mRNA secondary structure, mRNA stability and so on (Mitsuo, 1999; Tsai et al., 2008; Hunt et al., 2014). Our results showed that the protein level of BM group was lower than that of LP group.

On the one hand, the mRNA expression level and mRNA stability of BM group were higher and the mRNA secondary structure was more stable than that of the LP group, which may affect the initiation of IGF-1R protein translation and protein synthesis. Meanwhile, there is a strong heterogeneity between different haplotypes caused by synonymous mutations. For example, in the synonymous mutations found by Cheng et al. (2018) the group with high mRNA stability and protein stability may have a low expression level. But Capon et al. (2004) described that synonymous mutations with high mRNA stability showed high protein expression. On the other hand, IGF-1R proteins in LP group have low stability, and the half-life is shorter than that in BM group. However, this possible effect is not enough to offset the higher IGF1R synthesis in LP group based on our detection results. In addition, the process of protein synthesis is also affected by other factors and these influence needs to be further studied.

On the basis of the four synonymous mutations of IGF-1R ECD near the location of the IGF-1 binding domain, we speculated that the two haplotypes may have different binding affinities with IGF-1. The Co-IP experiment proved our conjecture that the relative binding rate of IGF-1 and IGF-1R significantly changed between the LP and BM groups. Moreover, the mechanisms underlying the relative difference in binding rates must be discussed. We considered that the number of receptors on the cell membrane surface changed. Previous studies found that the mutations in the extracellular domain of receptors can change their expression on the cell membrane surface (Ip et al., 2018). This change may be the reason for the difference in the ligand-binding rates of the different haplotypes of *Igf1r*. We detected a significant difference in the relative membranal expression rates of the two haplotypes *Igf1r*. We speculated further that the subtle changes in protein conformation of IGF-1R may affect transport, thereby decreasing the expression of receptors on the cell membrane. Numerous studies reported that genetic mutations can affect the changes in protein conformation. Atomic-level techniques, such as nuclear magnetic resonance or X-ray crystallography, can detect protein conformations. However, a study utilizing such techniques for detecting synonymous mutations has not been conducted yet. Subtle differences in protein conformations can also be inferred via differential immunodetection (Hunt et al., 2014). Proteins of different conformations have different binding efficiencies with a specific conformational sensitivity antibody. Thus, detecting conformation differences of proteins caused by synonymous mutations is a feasible method. In this study, conformation detection revealed that the synonymous mutation of IGF-1R changed the receptor’s binding affinity with the IGF-1R antibody. We speculated that it might be the synonymous mutation that affected the conformation of IGF-1R protein. The difference in conformations of the two haplotypes of *Igf1r* confirmed our prediction. We considered the difference as one of the reasons for the observed difference in binding capacity with IGF-1. These differences directly change the binding rates of IGF-1R and IGF-1. Moreover, these differences affect the transduction of intracellular signaling pathways and the differentiation of osteoblasts.

Owing to the extensive mechanisms of synonymous mutations and their random degrees of influence, several uncertainties and contingencies in the study of synonymous mutations remain. Therefore, the effects of synonymous mutations on the functions of specific genes must be explored. On the basis of the theory of codon preference, we observed that large pigs preferred to use common codons, whereas small pigs preferred rare codons. In combination with these results and the findings of previous studies, rare codons may lead to extreme translational pauses, provoke protein co-translation folding mechanisms, and change protein conformations and binding capacity (Tsai et al., 2008; Nissley et al., 2016). Synthesis of the receptor caused by synonymous mutations in the human dopamine receptor D2 (*Drd2*) alters mRNA stability (Duan et al., 2003). In turn, this alteration may affect the continuity of translation and even cause translational pause (Mitsuo, 1999). Synonymous mutations and ribosome stalling may lead to altered folding pathways. Although the conformational and functional differences between native and alternative states may be minor, subtle differences in conformations caused by synonymous mutations in *Mdr1* (*P*-glycoprotein) alter the structure of the substrate and inhibitor interaction sites (Tsai et al., 2008; Fung et al., 2014). Thus, even slight differences caused by synonymous mutations may affect physiological functions. In this study, we found that the difference in binding affinity with IGF-1 caused by the four *Igf1r* synonymous mutations. Another study showed that a synonymous mutation of its ligand IGF-1 has similar effects (Cheng et al., 2018), suggesting that these findings are not isolated cases. Meanwhile, the codon preference differences between the large and miniature pigs we observed suggested that codon usage may be a factor regulating body size. Further studies are warranted to prove or falsify this hypothesis.

## Conclusion

We detected two haplotypes formed by four synonymous mutations in IGF-1R ECD from pigs with different body size traits. These haplotypes may affect *Igf1r* transcription and translation, IGF-1R protein conformation, and IGF-1 interactions, resulting in differences in osteoblast differentiation and mineralization. Furthermore, the linkage effects of these synonymous mutations may be involved in the formation of body size in miniature pigs. We explored the functions of potentially valuable synonymous mutations. Our results provide a theoretical basis for the formation of body size in miniature pigs.

## Data Availability Statement

The original contributions presented in the study are included in the article/supplementary material, further inquiries can be directed to the corresponding author.

## Author Contributions

LH, CW, and SW conceived and designed the experiments. CW and SW performed the experiments. LH, CW, SW, SL, YC, HG, RY, TF, GL, XS, and JS assessed the experiments and provided the data analysis. CW and SW wrote the manuscript. All the authors read and approved the manuscript.

## Conflict of Interest

The authors declare that the research was conducted in the absence of any commercial or financial relationships that could be construed as a potential conflict of interest.

## Footnotes

1 https://zlab.bio/guide-design-resources

2 https://blast.ncbi.nlm.nih.gov/Blast.cgi
